# The use of vagal manoeuvres in narrow complex tachyarrhythmias in primary care

**DOI:** 10.4102/safp.v64i1.5413

**Published:** 2022-01-26

**Authors:** Shane D. Murphy, Michele Torlutter

**Affiliations:** 1 Department of Family Medicine, Faculty of Health Sciences, University of the Witwatersrand, Johannesburg, South Africa

**Keywords:** family medicine, primary care, rural medicine, supraventricular tachycardia, vagal manoeuvre

## Abstract

Supraventricular tachydysrhythmias (SVTs) are a common presenting complaint, with a national prevalence of 3/1000 persons. While most commonly stable, prolonged paroxysms can deteriorate into haemodynamically unstable subtypes or ventricular dysrhythmias. Early recognition with appropriate management is critical to reducing the morbidity associated with this condition. The American Heart Association holds that vagal manoeuvres are a first-line therapy in the management algorithm of stable SVTs. However, they state that no clear recommendations can be made around which manoeuvre to use, highlighting that future research should examine the efficacy and safety profiles of the various manoeuvres. In the South African primary care setting, clinicians must be at the forefront of pragmatic management strategies in the face of resource limitations, such as the unavailability of adenosine – a second-line therapy when vagal manoeuvres fail. In this article, we begin with a case study and review the literature around vagal manoeuvres.

## Case study

### Presentation

A 35-year-old man presented to our centre during a weekend shift. He reports a 2-h history of experiencing palpitations and anxiety. The symptoms had begun at around 4 o’clock in the afternoon after a Sunday lunch with his family. He had drunk four units of alcohol over a 3-h period and smoked three cigarettes earlier that day.

### Past medical

He reported two previous episodes at 29 years and 33 years of age. During the previous episodes, inpatient workup had ruled out any metabolic and/or endocrinological abnormalities, as well as structural heart disease (by electrocardiography [ECG] and echocardiography). He is currently awaiting his cardiology outpatient appointment date. He has no chronic medical illnesses and had an appendectomy at 13 years of age. There was no family history of cardiac disease.

### Social

He reports occasional alcohol use of around four units of alcohol per weekend, with no use during the week. He is not a regular smoker, smoking up to five cigarettes in a month. The patient did not use any illicit substances and drank three cups of coffee per day. He resides in a four-room house with his life partner of 6 years. Both are employed, have stable jobs, and he reports no psychosocial stressors.

### Examination

The patient’s observations were as follows:

- Blood pressure (BP): 135/85
- Pulse: 215 beats per minute
- Saturation: 96% (room air)
- Respiratory rate: 22

Examination reveals good peripheral perfusion with a regular tachycardia. Air entry was equal bilaterally, without adventitious sounds. There was no lower limb oedema or jugulovenous distension. The liver was not palpable.

**FIGURE 1:**
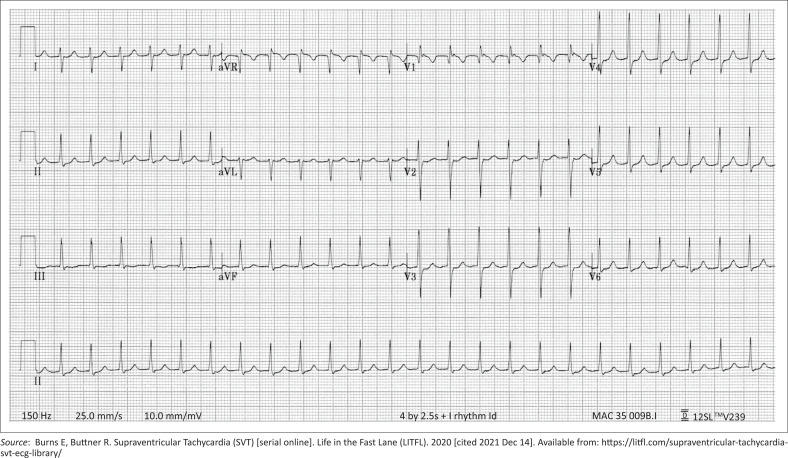
Electrocardigraph (ECG) example of narrow complex supraventricular tachydysrhythmias.

This patient was assessed as having a stable supraventricular tachycardia (SVT). He was moved to the high care area in the emergency centre, placed on monitors, baseline bloods were drawn and an ECG was done that showed a regular, narrow complex tachycardia, similar to the ECG in Figure 1.^1^

A modified Valsalva manoeuvre was performed and normal sinus rhythm was restored. A post-reversion ECG was unremarkable.

## Background

Tachydysrhythmias are defined as abnormal heart rhythms with a ventricular rate above 100 beats per minute.^2^ Supraventricular tachydysrhythmias are dysrhythmias that originate at, or above, the level of the His-Purkinje system^3^ from either atrial tissue or the atrioventricular (AV) junction:

- Atrial tissue
  ■ Sinus tachycardia
  ■ Inappropriate sinus tachycardia
  ■ Sinoatrial nodal re-entrant tachycardia (SNRT)
  ■ Atrial fibrillation
  ■ Atrial flutter
  ■ Intra-atrial re-entrant tachycardia
- Atrioventricular junction
  ■ Atrioventricular nodal re-entrant tachycardia (AVNRT)
  ■ Atrioventricular re-entrant tachycardia (AVRT)
  ■ Junctional tachycardia

Supraventricular tachydysrhythmias are most commonly narrow complex tachydysrhythmias but can have broad complexes when there is pre-existing aberrant conduction (e.g. bundle branch blocks), tachycardia-induced aberrancy (physiological), or, in the case of an accessory pathway, with antidromic conduction.

While local literature on the incidence of SVT is sparse, evidence shows that the national prevalence approximates international figures at around 3/1000 persons.^4^ The majority of SVTs are because of re-entry pathways, without the presence of underlying cardiac disease.^3^ Common symptoms include syncope/presyncope, palpitations, shortness of breath, diaphoresis, chest pain and lightheadedness/dizziness.

## An approach

Most cases of SVT are stable unless there is an underlying cardiac failure, ischaemia or other co-morbidities (such as sepsis or toxin ingestion).

Determining the haemodynamic stability of the patient is the first step in evaluation. A useful mnemonic for this is *HASIA*:

- Hypotension
- Altered mentation
- Shock
- Ischaemic chest pain (poor coronary perfusion pressure)
- Acute pump failure (pulmonary oedema)

If any of these cues are present, attempt to rapidly rule out sinus tachycardia^3^ (and treat the underlying cause). If this is ruled out, immediate synchronised cardioversion according to Advanced Cardiac Life Support principles is warranted.

Provided there are no emergent signs or symptoms that warrant synchronised cardioversion, we regard the SVT as stable and proceed with the evaluation of the SVT:^3^

- Is it regular or irregular?
- Are P waves discernible? If so, what is their morphology?

Perform the Valsalva maneuver!!:

- The American Heart Association describes vagal manoeuvres as a class one recommendation (level of evidence B-R) for first-line therapy of stable SVTs,^3^ although they note that vagal manoeuvres require further refinement to establish their effectiveness and safety.^3^

### Which vagal manoeuvre is best?

Studies have recurrently shown the Valsalva manoeuvre (VM) as superior to other options in adults:

- Valsalva success rate: 20% – 55%^5,6^
- Carotid sinus massage success rate: 5% – 33%^6,7^
- Diving reflex (cold ice immersion) success rate: 17%^8^

The diving reflex can be technically challenging and is not frequently employed in adults. In children, eliciting the diving reflex by use of a cold water bucket is supported as a first choice intervention by high quality evidence.^3^ While the carotid sinus massage is the simplest to perform, studies have reported iatrogenic peril (thromboembolic stroke from dislodged carotid atheroma) in around 1% of cases.^7^ The greater effectiveness and improved risk profile of the VM have seen it become a first choice vagal manoeuvre for the treatment of stable SVT in adult patients There are several challenges around the use of adenosine in the primary care setting that limit its use (see Box 1).

BOX 1 What about adenosine?

The pharmacokinetics of adenosine make it a highly favourable agent^5^:

- Rapid onset
- Short half-life (< 10 seconds)
- Highly selective for adenosine-1 receptor:
*A1 receptor – induces asystole by inhibiting adenylyl cyclase and reducing intracellular cAMP, which hyperpolarizes cardiac cells by increasing K+ efflux (in turn inhibiting Ca2+ and B-agonist receptors)* ^4^
- Rapid intracellular metabolism

However, the adverse effects of adenosine include a high incidence of new and transient cardiac arrhythmia (up to 55%), dyspnoea (12% – 28%), chest tightness (40%), dizziness (< 12%), headache, facial flushing (44%), nausea and an ‘electric shock’ sensation.^6^

Contraindications include hypersensitivity, high-grade heart block, sick sinus rhythm, bronchoconstrictive or bronchospastic disease, including asthma.^6^

The success of adenosine is dependent on several technical factors such as the proximity of venous access to heart, elevation of the limb and the rapid use of saline flush with a three-way stop cock.

Note: In South Africa, adenosine is not available at community health centres or clinics. It is not found in the essential drug list and is not mentioned as a step in the management of tachydysrhythmias in the standard treatment guidelines.^6^

A recent modification to the VM was employed in the landmark REVERT trial (see Tables 1 and 2), which showed increased sinus reversion rates (compared to the conventional VM) and no increase in harm Subsequent trials have validated these findings.^11^

**TABLE 1:** The REVERT trial – Primary and secondary outcomes.

Outcome	Standard VM				Modified VM				Effect size				
	*n* (214)	%	Median	IQR	*n* (214)	%	Median	IQR	OR	95% CI	Median	IQR	*p*
Presence of sinus rhythm at 1 min	37	17	-	-	93	43	-	-	3.7	2.3–5.8	-	-	< 0.0001
Adenosine needed	148	69	-	-	108	50	-	-	0.45	0.30–0.68	-	-	0.0002
Any other emergency anti-arrhythmic needed	171	80	-	-	121	57	-	-	0.33	0.21–0.51	-	-	< 0.0001
Discharged home from emergency room	146	68	-	-	134	63	-	-	0.79	0.51–1.21	-	-	0.28
Any adverse events	8	4	-	-	13	6	-	-	1.61	0.63–4.08	-	-	0.32
Time spent in emergency room (h)	-	-	2.83	1.05–3.62	-	-	2.82	1.95–3.77	-	-	0.90	0.75–1.10	0.31

*Source*: Adapted from Appelboam A, Reuben A, Mann C, et al. Postural modification to the standard Valsalva manoeuvre for emergency treatment of supraventricular tachycardias (REVERT): A randomised controlled trial. Lancet. 2015;386(10005):1747–1753. https://doi.org/10.1161/01.CIR.98.24.2716

VM, Valsalva manoeuvre; CI, confidence interval; IQR, interquartile range; OR, odds ratio.

**TABLE 2:** The Revert trial – Frequency of adverse events.

Adverse event	Standard VM (*n* = 214)	Modified VM (*n* = 214)
Increased heart rate	4	3
Hypotension or light-headedness	3	3
Electrocardiograph captured events	2	3
Other†	0	5
Musculoskeletal pain‡	0	4

*Source*: Adapted from Appelboam A, Reuben A, Mann C, et al. Postural modification to the standard Valsalva manoeuvre for emergency treatment of supraventricular tachycardias (REVERT): A randomised controlled trial. Lancet. 2015;386(10005):1747–1753. https://doi.org/10.1161/01.CIR.98.24.2716

VM, Valsalva manoeuvre.

† , Transient headache (*n* = 2); shortness of breath (*n* = 1); cyanosis (in different patients; *n* = 1);

‡ , Transient chest wall pain on straining.

### The physiology behind the Valsalva manooeuvre

**FIGURE 2:**
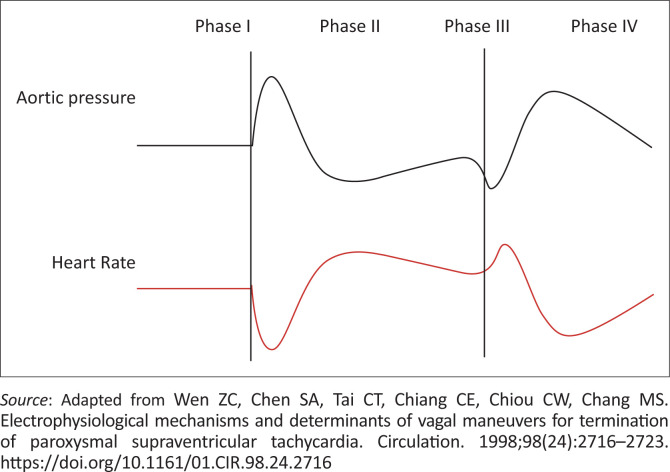
The physiology of the Valsalva manoeuvre.

Conceptually, the aim of the VM is to induce a cascade of four phases vagotonic fluxes that incrementally increase until sufficient vagal tone to induce AV nodal blockade is achieved (see Figure 2).

Phase I:

- Increased intrathoracic pressure – systolic blood pressure (SBP) rises (> 15 mmHg) following initiation of Valsalva (10 s)
  ■ Triggers baroreceptor reflex (either at a carotid bifurcation or within aortic arch)
  ■ Afferent impulses via glossopharyngeal (carotid) or vagus (aortic) nerve to vagal nuclei within nucleus tractus solitarius within the medulla

Phase II:

- Decreased venous return; increased systemic arterial pressure; blood pressure return to (just below) normal while a strain is maintained
  ■ Efferent impulses from vagal nuclei descend both right and left vagal nerves to heart, lungs and gastrointestinal tract.
  ■ Right vagus nerves stimulate sinoatrial (SA) node – decreased firing
  ■ Left vagus innervates av node – decreased conduction

Phase III:

- Decreased intrathoracic pressure. Blood pressure drops when strain is released.

Phase IV:

- Rapid increase in BP as sympathetic surge response to decreased systolic pressure.
- The response to the hypotension induced by the VM is the final means by which we induce a strong vagal tone to terminate the tachydysrhythmia.

The purpose of a vagal manoeuvre is to impact cardiac physiology. The desired effects are mostly achieved in phases I and IV:^10^

- SA node – slowed impulse generation in SA node.
- Atria – normal conduction velocity; prolonged refractory period.
- AV node – decreased conduction in AV node with prolonging of the refractory period.
- Ventricles – decreased inotropy; normal conduction in His-Purkinje system.

When should we use the VM?

- Therapeutic – Haemodynamically stable patients: first-line therapy of SVT.
- Diagnostic – May help distinguish SVT from VT by slowing conduction.

**FIGURE 3:**
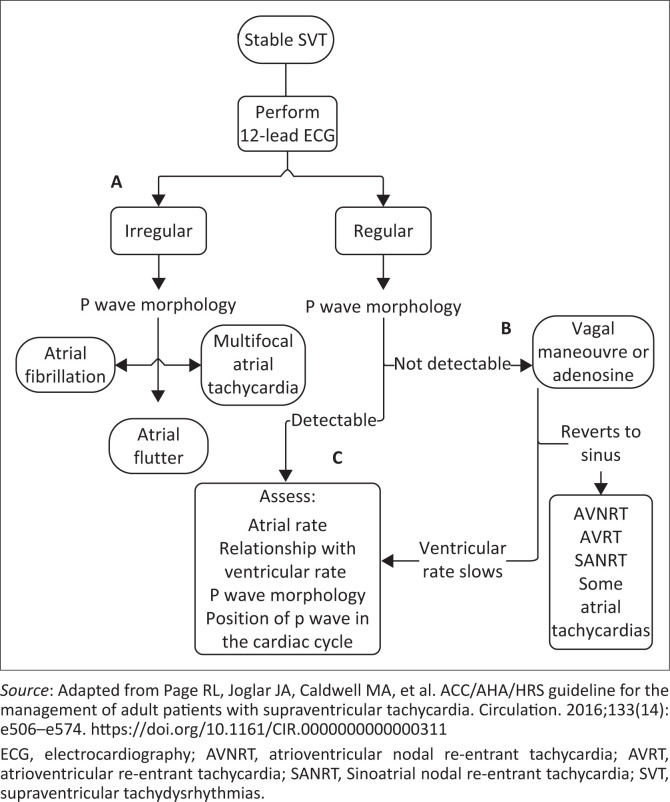
Evaluation of supraventricular tachydysrhythmias.

As mentioned, the most profound vagotonic effects are induced at the SA and AV nodes, and hence these rhythms undergo the highest reversion success rates. Figure 3 provides an approach to the patient presenting with stable SVT and three clinical scenarios (A, B and C):

- **A** – Vagal manoeuvres can terminate tachycardias of atrial origin, but they have a lower success rate and care must be taken to not perform AV node blockade in wide-complex tachycardias or if the patient has a known accessory pathway (risk of inducing ventricular fibrillation).
- **B** – Vagal manoeuvres are most successful in AV nodal-dependent SVTs (AVRT and AVNRT). At this stage, the clear dichotomy between therapeutic effect (reversion to sinus rhythm) and diagnostic application (slowing the rate to discern the dysrhythmia present and determine the best management approach) can be noted.
- **C** – If p waves are present and can be characterised, either can vagal manoeuvres be tried or an effective therapeutic agent selected based on the type of rhythm.

### How to perform the modified Valsalva

Equipment:

- 12 lead ECG placed on patient
- Cardiac monitoring – continuous
- Pulse oximetry
- Non-invasive BP monitoring – cycling frequently
- Intravenous access with fluids either running or immediately available
- Resuscitation trolley available
  ■ Airway
  ■ Oxygen
  ■ Crash cart with appropriate antiarrhythmic available and procedural sedation should cardioversion be required

Staff:

- Lead clinician
- Nursing assistant for monitoring
- If available, use an assistant to elevate legs

An informed, cooperative patient is paramount. The American Heart Association advocates teaching a patient to cough on command (vagotony from coughing terminates a bradycardia consequent to the Valsalva).^3^

Technique:^9^

- Attach all monitors, have support staff.
- Patient is seated in an upright seated position.
- Patient blows against a 10 mL syringe to move plunger (approximately 30–40 mmHg) for 10–20 s
- Immediately drop patient to supine position
- Simultaneously elevate patient’s legs to 45° – 90°
- Hold for 45–60 s
- Repeat if necessary

The ‘modification’ of the Valsalva by dropping the head of the patient, elevating the legs, and finally lifting the head of the patient accentuates the physiological effects of the Valsalva manoeuvre – increasing the likelihood of success.

### Clinical bottom line and applicability to the patient

Supraventricular tachydysrhythmias are the most common tachyarrhythmias with an incidence of 3/1000 persons. Most SVTs are benign conditions without underlying structural heart pathology. However, their propensity to deteriorate into unstable SVTs or ventricular fibrillation mandates early and effective treatment. The modified VM is the most effective vagal manoeuvre with minimal documented adverse effects and should be used as the first-line therapy in the management of SVT.

The patient in our case study was a 35-year-old man who presented with a recurrent, stable AVNRT in the absence of any underlying structural heart disease. The effective management of his AVNRT avoided unnecessary admission to a higher level of care while awaiting his outpatient cardiology appointment. This case highlights the value of evidence-based practice in the primary care setting to provide appropriate care while avoiding unnecessary medical intervention with its associated costs and potential harm.
